# Ununited Fracture Cured by Seton

**Published:** 1829-10-01

**Authors:** 


### WINCHESTER COUNTY HOSPITAL.

### UNUNITED FRACTURE CURED BY THE SETON.*

The troublesome and frequently indomitable character of many cases of non-union of bone, renders any plan of treatment that promises to be even generally successful a desideratum of much importance. We believe that properly regulated pressure has been found the most useful in the long run, and Mr. Amesbury's apparatus effects this object better perhaps than any other which has hitherto been invented. But pressure, however and whenever applied, will sometimes, indeed not unfrequently, fail, and it then becomes useful to determine what other measure shall be had recourse to. Next to pressure we would place the seton in the scale of valuable methods of treatment, at least we conceive that published cases establish this opinion. It has often been productive of no sort of good, but in many instances, on the other hand, it has answered completely and effected a cure. Mr. Henry Lyford has published an interesting example of the efficacy of the seton in our Provincial Cotemporary, and we think we shall do right by giving it a more extensive circulation in our columns, than the comparatively narrow range of a local journal will procure it.

The subject of the case was a boy, who broke his right thigh eleven months before his admission into hospital under Mr. Lyford. The fracture had been attended to by a surgeon in the usual mode, but had never united, although the dressings were continued up to the period of his application. On examination, an oblique fracture was discovered rather above the centre of the femur, where so much motion existed that the two ends of bone could readily be made to form an obtuse angle with each other. The foot and leg, which were œdematous, were much everted, and the broken limb an inch and a quarter permanently shorter than its fellow. The constitution appeared to be extremely languid, the appetite was bad, the bowels confined. A large blister was applied on each side of the thigh near the fracture, and strengthening diet, with a mercurial aperient, and the compound steel mixture, were prescribed, but although the general health was improved, no ossific union whatever took place. Accordingly, on the 12th day after his admission, Mr. L. introduced the seton in the following manner. An incision, an inch and a half in length, being made through the integuments in the middle of the thigh, at the inner edge of the rectus muscle, the subjacent parts were completely divided down to the fracture. A long round seton needle, armed with a piece of tape well oiled, was then introduced between the fractured extremities of the bone, and protruded behind the vastus externus, through the skin, at the outer and posterior part of the limb. No hæmorrhage whatever ensued, the wounds were dressed with adhesive plaster, and the patient placed on his back in bed, with the leg and thigh supported by the double-inclined plane. The wounds were dressed on the third day, and decoction of bark, in addition to the previous strengthening diet, was prescribed. All went on well till the 13th after the operation, when the patient was attacked with frequent rigors, considerable pain and swelling in the

* Prov. Med. Gaz. No. 1.

thigh, general febrile disturbance, and an erysipelatous blush around the wounds. The seton was withdrawn, and a poultice applied, and calomel, opium, and an aperient draught were given, with the effect of gradually removing the constitutional affection. A high degree of inflammation, however, attacked the extremities of the bone, and the slightest motion of the limb, or indeed of the body, produced the most excessive pain in the fracture. Perfect rest, by means of the many-tailed bandage and splints, succeeded completely in arresting this, and, at the end of two months from the time of his admission, the patient was dismissed from the hospital cured. The limb was an inch and a quarter shorter than the other, which occasioned a limp in his gait.

---

